# Non-amyloidogenic effects of *α*2 adrenergic agonists: implications for brimonidine-mediated neuroprotection

**DOI:** 10.1038/cddis.2016.397

**Published:** 2016-12-08

**Authors:** Shereen Nizari, Li Guo, Benjamin M Davis, Eduardo M Normando, Joana Galvao, Lisa A Turner, Mukhtar Bizrah, Mohammad Dehabadi, Kailin Tian, M Francesca Cordeiro

**Affiliations:** 1 Glaucoma and Retinal Neurodegeneration Group, Department of Visual Neuroscience, UCL Institute of Ophthalmology, London EC1V 9EL, UK; 2 The Western Eye Hospital, Imperial College Healthcare Trust, London NW1 5QH, UK

## Abstract

The amyloid beta (A*β*) pathway is strongly implicated in neurodegenerative conditions such as Alzheimer's disease and more recently, glaucoma. Here, we identify the *α*2 adrenergic receptor agonists (*α*2ARA) used to lower intraocular pressure can prevent retinal ganglion cell (RGC) death via the non-amyloidogenic A*β*-pathway. Neuroprotective effects were confirmed *in vivo* and *in vitro* in different glaucoma-related models using *α*2ARAs brimonidine (BMD), clonidine (Clo) and dexmedetomidine. *α*2ARA treatment significantly reduced RGC apoptosis in experimental-glaucoma models by 97.7% and 92.8% (BMD, *P*<0.01) and 98% and 92.3% (Clo, *P*<0.01)) at 3 and 8 weeks, respectively. A reduction was seen in an experimental A*β*-induced neurotoxicity model (67% BMD and 88.6% Clo, both *P*<0.01, respectively), and *in vitro*, where *α*2ARAs significantly (*P*<0.05) prevented cell death, under both hypoxic (CoCl_2_) and stress (UV) conditions. In experimental-glaucoma, BMD induced ninefold and 25-fold and 36-fold and fourfold reductions in A*β* and amyloid precursor protein (APP) levels at 3 and 8 weeks, respectively, in the RGC layer, with similar results with Clo, and *in vitro* with all three *α*2ARAs. BMD significantly increased soluble APP*α* (sAPP*α*) levels at 3 and 8 weeks (2.1 and 1.6-fold) *in vivo* and *in vitro* with the CoCl_2_ and UV-light insults. Furthermore, treatment of UV-insulted cells with an sAPP*α* antibody significantly reduced cell viability compared with BMD-treated control (52%), co-treatment (33%) and untreated control (27%). Finally, we show that *α*2ARAs modulate levels of laminin and MMP-9 in RGCs, potentially linked to changes in A*β* through APP processing. Together, these results provide new evidence that *α*2ARAs are neuroprotective through their effects on the A*β* pathway and sAPP*α*, which to our knowledge, is the first description. Studies have identified the need for *α*-secretase activators and sAPP*α*-mimetics in neurodegeneration; *α*2ARAs, already clinically available, present a promising therapy, with applications not only to reducing RGC death in glaucoma but also other neurodegenerative processes involving A*β*.

Glaucoma is a major cause of visual impairment worldwide and is characterised by optic neuropathy and visual field loss. Retinal ganglion cell (RGCs) apoptosis is considered an early hallmark of glaucoma^1^ and raised intraocular pressure (IOP) is presently the only modifiable risk factor.^2^ As a proportion of glaucoma patients continue to lose vision despite effective IOP control,^3^ IOP-independent risk factors are increasingly thought to have a role in glaucoma pathology.

Amyloid beta (A*β*), the major constituent of senile plaques in Alzheimer's disease (AD), has recently been implicated in glaucoma pathology.^4, 5^ A*β* is associated with abnormal processing of amyloid precursor protein (APP). APP can be cleaved either by *α*-secretase via the non-amyloidogenic pathway, producing soluble APP*α* (sAPP*α*), or *β*-secretase producing sAPP*β*, and A*β*, via the amyloidogenic pathway.^6^ Using rodent glaucoma models, the amyloidogenic pathway has recently been identified as a target for the development of novel neuroprotective glaucoma therapies.^4, 5^ Here, A*β* deposition was found to induce RGC apoptosis, a finding supported by a study on glaucoma patients reporting reduced A*β* concentrations in the vitreous.^7^ A*β* may therefore be important in the stress–response to glaucomatous neurodegeneration and offers a novel therapeutic target.^4^

Brimonidine (BMD), Clonidine (Clo) and Dexmedetomidine (Dex) are *α*2 adrenergic receptor agonists (*α*2ARAs). Apraclonidine, a para-amino derivative of Clo, is a topical *α*2ARA^8^ routinely used in the clinic to reduce IOP spikes induced by neodymium:YAG laser treatment for posterior capsule scarring after cataract surgery.^9^ However, the reduced activity of Apraclonidine in controlling IOP with chronic usage^10^ coupled with increased risk of follicular conjunctivitis,^11^ renders it unsuitable for long-term glaucoma management. BMD was introduced as an IOP-lowering agent; however, increasing experimental evidence suggests it also has IOP-independent neuroprotective activity.^12^ This was clinically demonstrated in a prospective, randomised-controlled study where BMD was reported to significantly preserve visual field in low-tension glaucoma patients compared with the beta-blocker timolol.^13^ Both Clo and Dex are used as anaesthetics,^14^ Clo is used to treat migraine, hypertension and menopausal flushing,^15^ and Dex for sedation during intensive care.^16^ *In vivo* studies have demonstrated Clo and BMD to have retinal neuroprotection^17, 18, 19^ with functional benefits,^20, 21^ and Dex to have neuroprotection against cerebral ischaemia,^22^ excitotoxicity^23^ as well as in a model of traumatic brain injury.^24^

Although *α*2A_A_ and *α*2A_B_ receptors have been identified in the RGC layer (RGCL) of the inner retina,^25^ the mechanisms by which *α*2A agonists exert neuroprotection are not well-established. Various pathways have been proposed, including cyclic adenosine monophosphate (cAMP) reduction,^26^ NMDA receptor neuromodulation,^21^ increasing cell survival proteins p-Akt and bcl-2 (ref. 27) and neurotrophic factor expression.^22^ The present study seeks to delineate the mechanisms of *α*2A-mediated neuroprotection using glaucoma-related *in vivo* and *in vitro* models, and investigate the involvement of the A*β*-pathway.

## Results

### *α*2ARAs are neuroprotective against retinal neuronal death *in vitro* and *in vivo*

**Figure 1:**
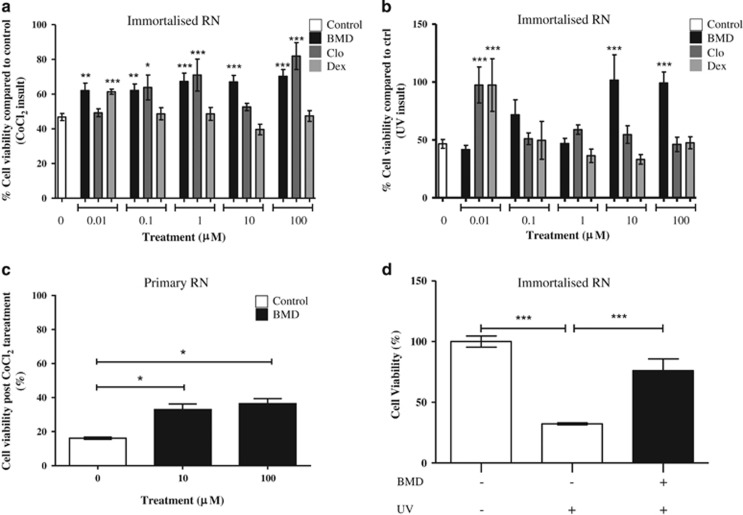
*α*2 agonists are neuroprotective against RGC death *in* *vitro. α*2 agonists' effects on neuroprotection were investigated *in* *vitro* using the MTT assay in immortalised RNs against CoCl_2_ (hypoxic) and UV (stress) insults; against 250 *μ*M CoCl_2_ BMD showed significant neuroprotection at all concentrations tested, Dex showed protection at 0.01 *μ*M and Clo was neuroprotective at 0.1, 1 and 100 *μ*M (**a**). Against the insult of UV, BMD was protective at 100 and 10 *μ*M and Clo and Dex were both protective at 0.01 *μ*M (**b**). The protective effects of BMD at 10 and 100 *μ*M against CoCl_2_ were reproduced in mouse-derived primary mixed retinal cultures (**c**). In immortalised RNs, BMD (10 *μ*M) was found to be protective against an UV-light insult of 80 j/cm^2^ (**d**). All experiments were carried out in triplicate. All means±S.E.M.; * *P*<0.05, **P<0.01, ***P<0.001

Retinal neuronal cells (RNs) were pre-treated for 24 h with BMD, Dex or Clo before insulting with the hypoxia-mimic cobalt chloride (CoCl_2_)^28^ or UV light to induce neurotoxicity, based on previously determined IC_50_ doses.^29^ CoCl_2_ induced a decrease in cell viability in both primary and immortalised cell types, which was significantly reduced with BMD at all concentrations assessed (0.1–100 *μ*M, *P*<0.01 Figures 1a and c), whereas Clo was protective at 0.1, 1 and 100 *μ*M (*P*<0.05), whereas Dex was effective at 0.01 *μ*M (*P*<0.001, Figure 1a).

BMD significantly increased cell viability of UV-insulted cells at 10 and 100 *μ*M (*P*<0.001, Figure 1b). Clo and Dex were neuroprotective at 0.01 *μ*M (*P*<0.001 and *P*<0.001, respectively, Figure 1b). BMD reduced UV-induced RN death in a dose-dependent manner with an IC_50_ value of 64±14 *μ*M. At a peak activity of 1 mM, BMD significantly increased cell viability against UV insult (76.1% compared with 32.2%, respectively, *P*<0.001, Figure 1d).

**Figure 2:**
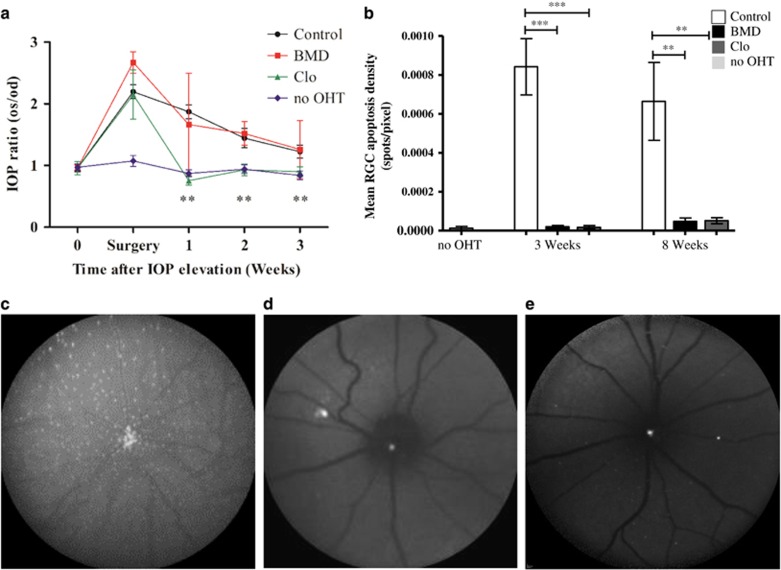
*α*2 agonists are neuroprotective against RGC death *in vivo.* (**a**) Rats had IOP surgically elevated in one eye (OHT os) in an established model of ocular hypertension (OHT). IOP was significantly increased in untreated control (*n*=10) and systemic BMD-treated groups (*n*=10) compared with no-OHT (contralateral eyes), up to 3 weeks after surgery. In comparison, systemic Clo treatment significantly reduced IOP (*n*=10). (**b**) *α*2A agonist treatment (BMD, Clo) significantly decreased RGC apoptosis compared with untreated controls at 3 (43-fold and 50-fold reduction for BMD and Clo, respectively) and 8 (14-fold and 13-fold reduction for BMD and Clo, respectively) weeks after surgery. (**c**–**e**) Representative DARC images showing the *in vivo* retinal image of an untreated OHT (**c**) compared with BMD (**d**) and Clo-treated (**e**) rats at 3 weeks after IOP elevation. Each white spot represents an individual retinal ganglion cell undergoing apoptosis that is positive for fluorescently labelled annexin A5. (**a**) and (**b**) show means±S.E.M.; **P<0.01, ***P<0.001

Effects of systemic *α*2A agonists BMD and Clo were examined *in* *vivo* using an ocular-hypertensive (OHT) rat model.^30^ Comparison of the IOP profile between untreated OHT control and no-OHT groups showed OHT surgery produced a significant increase in IOP up to 3 weeks post surgery (*P*<0.05, Figure 2a). Systemic Clo treatment lowered IOP at 1, 2 and 3 weeks compared with the untreated OHT group (all *P*<0.01), whereas systemic BMD had no effect on IOP (Figure 2a), as expected.^31^

Significantly, more apoptotic RGCs were observed at 3 and 8 weeks in the untreated OHT model compared with the no-OHT control eyes (*P*<0.01; Figure 2b), with peak RGC apoptosis occurring at 3 weeks, consistent with previous findings.^1, 4^ Both BMD and Clo significantly reduced RGC apoptosis compared with untreated controls at 3 weeks, by 97.7% and 98%, respectively (*P*<0.001), and at 8 weeks (92.8% and 92.3%, *P*<0.01), as shown in Figures 2b–e. No significant difference was observed between 3 and 8 weeks BMD and Clo-treated groups (*P*>0.05, Figures 2b–e). BMD's anti-apoptotic effect was found to coincide with increased levels of the cell survival protein P-Bad both *in vivo* and *ex vivo* (Supplementary data). In summary, these experiments demonstrate that *α*2ARA reduce levels of RGC apoptosis and cell death both *in vivo* and *in vitro*.

### The neuroprotective effect of *α*2ARAs is associated with modulation of A*β* in RNs *in vitro* and *in vivo*

**Figure 3:**
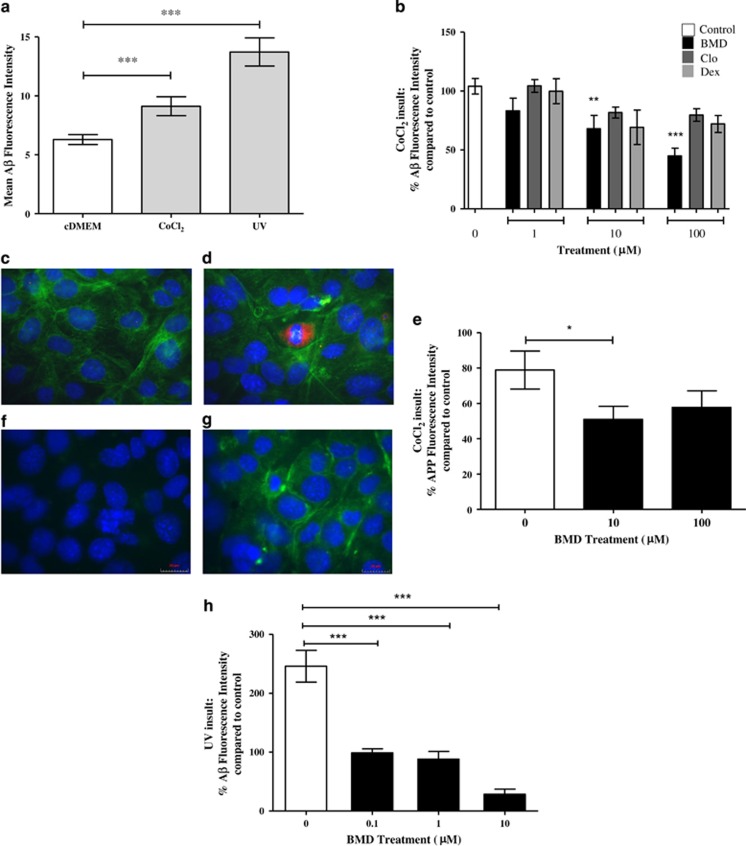
*α*2A agonists directly reduce retinal neuronal A*β* and APP levels in different *in vitro*stress models. *α*2 agonists' ability to reduce A*β* levels was next investigated *in vitro* in immortalised RNs using CoCl_2_ and UV insults, which significantly increased A*β* levels (**a**). A*β* levels were significantly reduced in CoCl_2_ samples when co-treated with 10 and 100 *μ*M BMD (**b**). All *α*2A treatments were associated with a reduction in A*β* levels at concentrations of 10 and 100 *μ*M, although BMD was found to be most effective. Representative images from CoCl_2_ insulted cells show BMD treatment (**c**) reduced A*β* (red) staining in RNs compared with CoCl_2_ only treatment (**d**); (*α*-tubulin staining (green), DAPI staining (blue)). APP levels were significantly reduced with 10 *μ*M BMD treatment against CoCl_2_ insult (**e**). Representative images from CoCl_2_ insulted RGCs show BMD treatment (**f**); reduced APP (green) staining in RGCs compared with CoCl_2_ only treatment (**g**); (DAPI staining (blue)). (**h**) Staining for A*β* induced by UV insult revealed a significant decrease in levels with treatment of 0.1, 1 and 10 *μ*M BMD. Experiments were carried out in triplicate. All data; means±S.E.M.; * *P*<0.05, **P<0.01, ***P<0.001

Previous studies suggest that glaucoma-related RGC apoptosis involved A*β*, and its therapeutic targeting was neuroprotective.^4^ The interaction of *α*2ARAs BMD, Dex and Clo with RN-associated A*β* was investigated *in vitro* with CoCl_2_ and UV-associated cell toxicity using immunocytochemistry. Both CoCl_2_ and UV significantly increased A*β* levels (*P*<0.001, Figure 3a) by 1.5- and 2.2-fold, respectively. Treatment with *α*2ARA on CoCl_2_ insulted cells significantly decreased A*β* detected when co-treated with 10 and 100 *μ*M BMD (*P*<0.01 and *P*<0.001, respectively), with a dose-dependent reduction in A*β* levels observed (20%(1 *μ*M), 34.7%(10 *μ*M) and 56.8%(100 *μ*M), Figures 3b–d). At the same concentrations, Clo and Dex treatments were associated with reductions in A*β* staining of; Clo: 0.3, 21.4, 23.4% and Dex: 4, 33.5 and 30.8%, respectively. In all, 10 and 100 *μ*M BMD treatment also significantly reduced APP detected, by 35.4 and 26.8% (Figures 3e–g; *P*<0.05). BMD treatment was more effective with lower concentrations on UV-induced A*β*, with 60.5, 64.5 and 81.4% decreases at 0.1 *μ*M, *1 μ*M and 10 *μ*M, respectively (*P*<0.001, Figure 3h).

**Figure 4:**
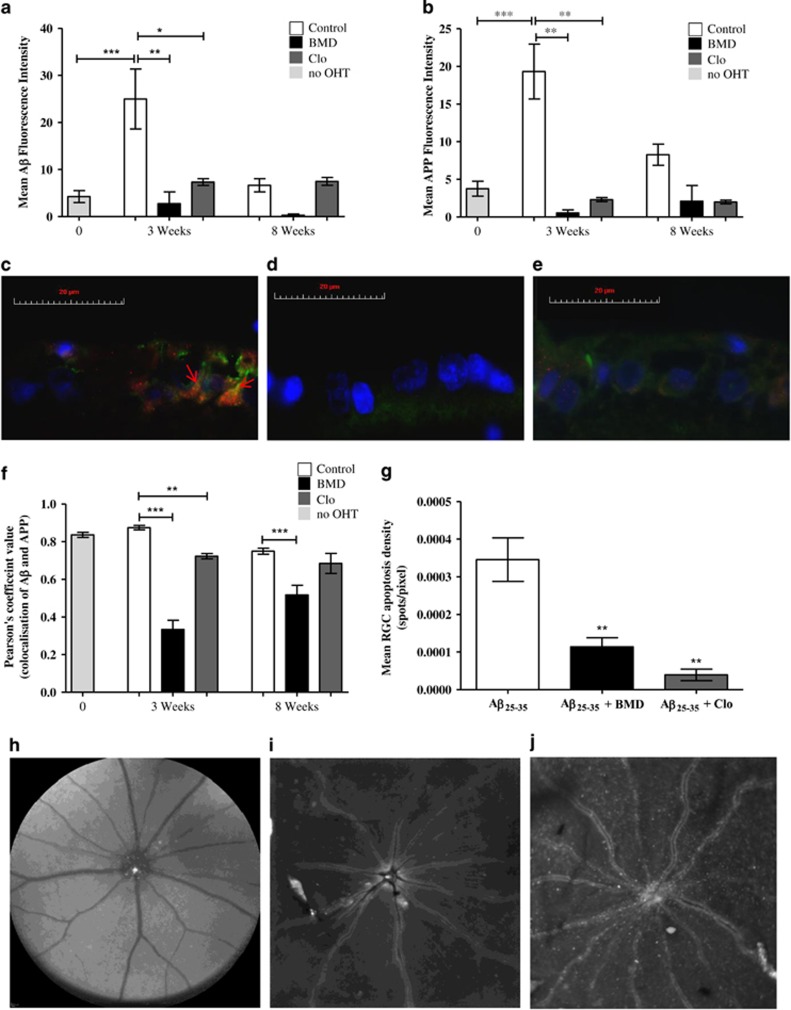
The neuroprotective effect of *α*2A receptor agonists is associated with modulation of A*β* *in vivo*. (**a**) Immunohistochemistry revealed a significant increase in A*β* in OHT eyes compared with the no-OHT control at 3 weeks post IOP elevation. BMD and Clo treatment significantly reversed this effect (BMD; ninefold decrease at 3 weeks and 25-fold decrease at 8 weeks, Clo; a 3.4-fold decrease at 3 weeks). (**b**) APP concentrations were similarly increased at 3 weeks in comparison with the no-OHT control. BMD and Clo treatment significantly reversed this effect with a 36-fold (BMD) and eightfold (Clo) reduction in APP levels in the RGCL at 3 weeks, and a fourfold reduction (BMD and Clo) at 8 weeks. Representative images of A*β* (red), APP (green) and nuclei (DAPI, blue) labelled RGCL of a 3 weeks OHT model in the absence (**c**) and presence of BMD (**d**) and Clo treatment (**e**), showing increased A*β* and APP colocalisation (highlighted by red arrows) in the untreated OHT compared with *α*2A agonist treated eyes (**d**, **e**). (**f**) *α*2A agonist treatment significantly decreased colocalisation of A*β* and APP detected at both 3 (BMD and Clo) and 8 weeks (BMD) compared with untreated OHT eyes. (**g**) Assessment of A*β* neurotoxicity was performed following intravitreal injection of A*β*_25–35_ *in vivo*. Both BMD and Clo significantly reduced levels of RGC apoptosis as detected using AlexaFluor 488 labelled annexin A5 to label apoptosing RGCs. (**h**) Representative *in vivo* image of *α*2A agonist treated A*β* model and histologically *ex vivo* (**i**), demonstrating a reduction in annexin labelled RGCs compared with untreated A*β* model (**j**). *In vitro* experiments were carried out in triplicate. All data; means±S.E.M. * *P*<0.05, **P<0.01, ***P<0.001

Consistent with previous *in vivo* findings,^4^ elevated levels of A*β* were observed in the RGCL 3 weeks post OHT model induction compared with no-OHT control (*P*<0.001, Figure 4a) associated with a significant increase in APP (*P*<0.001, Figure 4b).

Treatment of the OHT model with *α*2ARAs significantly reduced A*β* detected in the RGCL 3 weeks post IOP elevation (Figures 4a and c-e). BMD treatment was associated with a ninefold (3 weeks) and 25-fold (8 weeks) reduction in A*β* levels, whereas Clo induced a 3.4-fold (3 weeks) decrease (*P*<0.01 and *P*<0.05, respectively). A similar reduction in APP was observed in the RGCL with BMD treatment associated with a 36- (3 weeks) and a fourfold (8 weeks) reduction, and Clo an eightfold (3 weeks) and fourfold (8 weeks) reduction(*P*<0.01 in each case, Figures 4b–e). Furthermore, APP and A*β* showed greater colocalisation in the RGCL of the untreated OHT model (Figure 4c) compared with *α*2ARA-treated OHT model (Figures 4d and e); at 3 weeks (*P*<0.001 and *P*<0.01, BMD and Clo, respectively) and 8 weeks (*P*<0.001 BMD, Figure 4f). Although the Pearson's coefficient values are not significantly different in the OHT and the no-OHT controls, in the OHT controls there is a decreased A*β* (Figure 4a) and APP (Figure 4b) levels, as demonstrated by the ratio between the A*β* intensity and Pearson's coefficient value to in the no-OHT group being 5.10 compared with 28.6 in the OHT model at 3 weeks, and 8.87 at 8 weeks (Figures 4a and b). This change in colocalisation observed with *α*2ARA treatment is suggestive of altered processing of APP favouring the non-amyloidogenic pathway (Figure 4f), which is markedly different to the untreated no-OHT control, where less A*β* and APP was detected compared with the OHT model (Figures 4a).

To further investigate whether *α*2ARA therapy acted via an A*β*-dependent pathway, *α*2ARAs were administered into a previously characterised *in vivo* A*β*-induced RGC apoptosis model.^4^ BMD and Clo treatment significantly reduced RGC apoptosis in response to intravitreal administration A*β*_25–35_ (25 nM), compared with A*β*_25–35_ only controls (Figures 4g–j). RGC apoptosis was reduced by 67% (BMD, *P*<0.01) and 88.6% (Clo, *P*<0.01), respectively, suggesting a direct effect of *α*2ARAs on A*β*-induced RGC apoptosis.

### *α*2A agonists directly affect the non-amyloidogenic pathway and elicit neuroprotective activity through sAPP*α*

**Figure 5:**
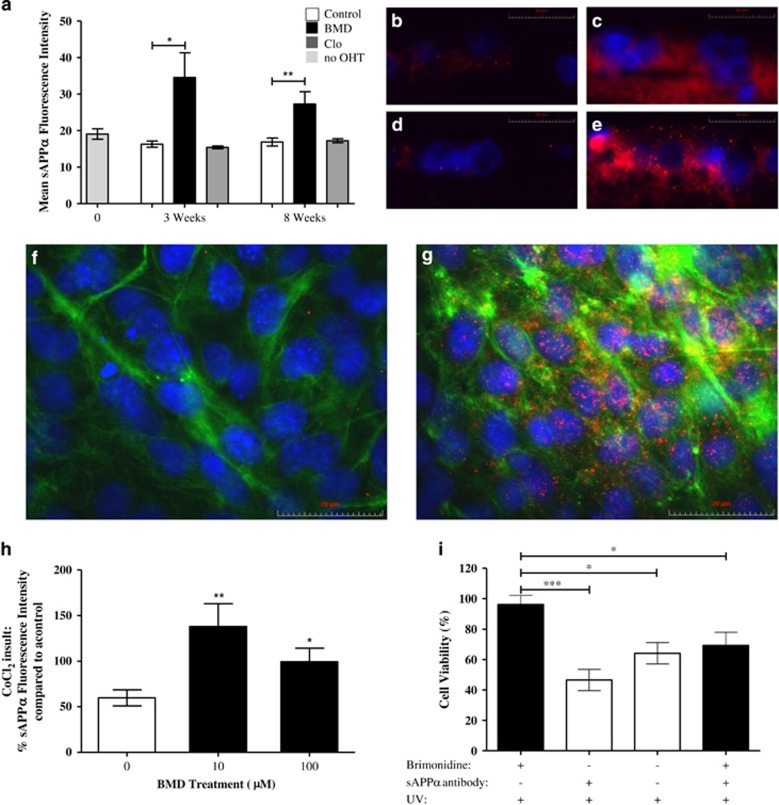
*α*2A agonists directly affect the non-amyloidogenic pathway and are neuroprotective through sAPP*α*. (**a**) Treatment with the *α*2A receptor agonist BMD significantly increased sAPP*α* levels at both 3 (2.1-fold) and 8 weeks (1.6-fold) compared with untreated control in the OHT model. (**b**–**e**) Representative retinal histological images of sAPP*α* (red) and nuclei (DAPI, labelled blue) of the RGCL of a 3 weeks (**b**, **c**) and 8 weeks. (**d**, **e**) OHT model in the absence (**b**, **d**) and presence (**c**, **e**) of BMD treatment, showing increased sAPP*α* staining with BMD. (**f**–**h**) sAPP*α* levels were significantly increased *in vitro* in the CoCl_2_ model with 10 and 100 *μ*M BMD treatment compared with control (**h**). Representative images from BMD and CoCl_2_ treated cells (**g**), and CoCl_2_ only treated cells (**f**) stained for sAPP*α* (red), *α* tubulin (green), DAPI (blue), show significantly increased sAPP*α* staining with BMD treatment. (**i**) sAPP*α* activity was next assessed using UV light to induce RGC death; the sAPP*α* antibody treatment significantly inhibited the neuroprotective effects of BMD against UV, suggesting that BMD is neuroprotective through sAPP*α*, promoting APP processing through the non-amyloidenic pathway. *in vitro* experiments were carried out in triplicate. Data are means±S.E.M. *P<0.05, **P<0.01, ***P<0.001

Having established *α*2ARA treatments reduce A*β*-associated-RGC apoptosis, we investigated whether these effects were mediated through changes to APP processing. Levels of sAPP*α* (a product of the non-amyloidogenic pathway^6^) were histologically assessed in the OHT model. sAPP*α* staining was not significantly different either at 3 or 8 weeks in the RGCL in untreated OHT and control eyes (Figure 5a). BMD treatment significantly increased sAPP*α* levels at both 3 and 8 weeks (2.1- and 1.6-fold increase, respectively) compared with untreated OHT controls (*P*<0.05 and *P*<0.01, Figures 5a–e). Clo treatment had no effect.

The effect of BMD on the non-amyloidogenic pathway was confirmed *in vitro* using the hypoxia mimetic CoCl_2_ to induce RN toxicity. A twofold significant increase in sAPP*α* levels was observed in response to 10 *μ*M and 100 *μ*M BMD treatment (*P*<0.01, *P*<0.05, respectively, Figures 5f–h).

Having previously established BMD treatment reduces A*β* levels in response to UV light induced toxicity, the effect of an sAPP*α* antibody on UV-insulted and BMD-treated cells was investigated. Although sAPP*α* antibody exposure caused no significant change in RGC viability after UV exposure, it significantly inhibited protection by BMD therapy (Figure 5i). This observation, coupled with data demonstrating that *α*2ARA treatment significantly reduced APP levels, suggests *α*2ARA mediated RGC neuroprotection is achieved through increased sAPP*α* through upregulation of non-amyloidogenic APP processing.

### The neuroprotective effect of *α*2ARAs via A*β*-related pathways involves modulation of the ECM

Laminin and MMP-9 expression have been implicated in A*β*-related pathways.^32^ The occurrence of RGC apoptosis in OHT is associated with a reduction in laminin in the RGCL at 3 months in the same OHT model.^33^ We therefore investigated whether extracellular matrix (ECM) modulation had a role in *α*2ARAs-mediated neuroprotection using the OHT model.

**Figure 6:**
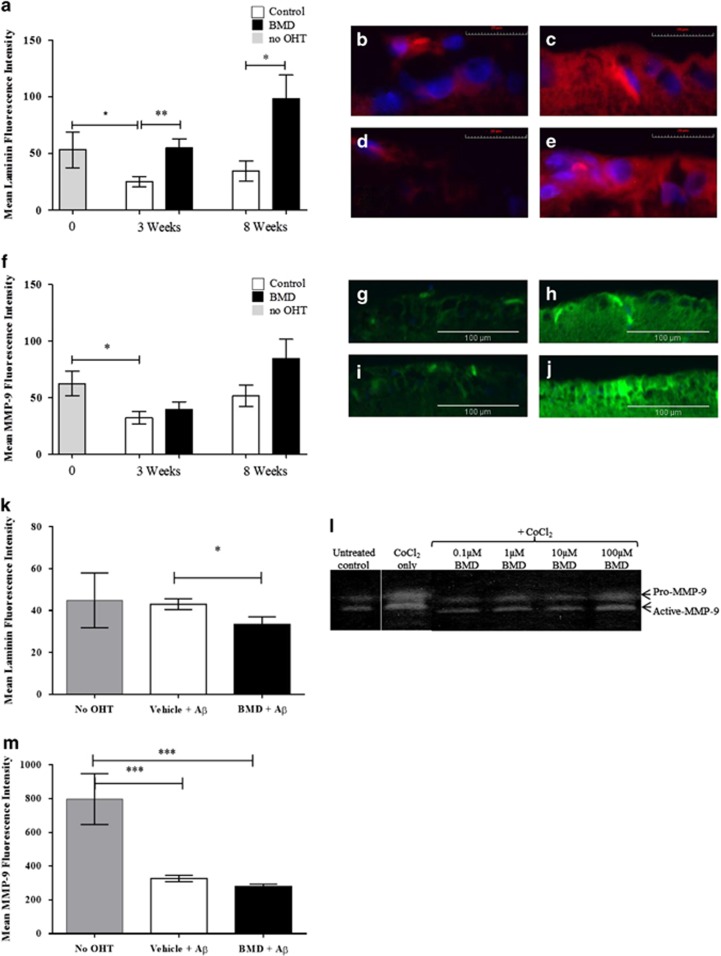
The neuroprotective effect of *α*2A agonist via A*β*-related pathways involves modulation of the extracellular matrix. (**a**) OHT surgery induced a significant reduction in laminin levels in the RGCL at 3 weeks compared with the no-OHT control. BMD treatment significantly increased levels of laminin at 3 (2.2-fold) and 8 weeks (2.9-fold) in comparison with the respective controls. (**b**–**e**) Representative images show low levels of laminin (red) in the RGCL of a 3 (**b**) and 8 (**d**) weeks OHT eye, compared with the same time points in BMD-treated OHT eyes (**c**, **e**), (DAPI, blue). (**f**) MMP-9 levels in the RGCL were significantly lower at 3 weeks in OHT eyes compared with the no-OHT control. (**g**–**j**) Representative images show the marked but non-significant increase in general (pro and active forms) MMP-9 levels (green) in the RGCL of BMD-treated OHT models at 3 (**h**) and 8 (**j**) weeks compared with OHT control eyes (**g**, **i**), (DAPI, blue). (**k**) In comparison, a different pattern was seen in the A*β* neurotoxicity model, where BMD treatment was associated with a significant reduction in laminin in the RGCL. (**l**) Further assessment of MMP-9 activity was performed using zymography on immortalised RN exposed to CoCl_2_, where BMD treatment appeared to substantially reduce MMP-9 activity in both pro- and active forms, although this effect was reduced at a high concentration (100 *μ*M). (**m**) BMD treatment did not significantly alter levels of MMP-9 in the RGCL of the A*β* apoptosis-inducing model, although levels were significantly different from no-OHT control. *in vitro* experiments were carried out in triplicate. Error bars±S.E.M. *P<0.05, **P<0.01, ***P<0.001

A twofold reduction in laminin was observed in the RGCL at 3 and 8 weeks post OHT induction compared with the no-OHT control eyes (*P*<0.05, Figure 6a). BMD treatment restored laminin levels in the OHT model RGCL at both 3 and 8 weeks (*P*<0.05, Figures 6a–e). A significant (*P*<0.05) twofold reduction in MMP-9 expression was observed in the RGCL 3 weeks post OHT induction but not at 8 weeks (Figures 6f–j). BMD treatment induced a marked but not statistically significant increase in MMP-9 levels at 8 weeks (Figure 6f). Laminin and MMP-9 expression was also investigated in the A*β*-inducing apoptosis model.^4^ BMD treatment was associated with a significant reduction in laminin deposition in the RGCL versus A*β*-treated controls, although there were no differences in comparison to the no-OHT control (unpaired *t*-test, *P*<0.05, Figure 6k).

To further investigate the effect of *α*2ARAs on MMP-9 expression, *in vitro* studies were performed on RNs insulted with CoCl_2_. Using zymography, CoCl_2_ increased both pro- and active MMP-9 (Figure 6h, lane 2), compared with untreated cells (lane 1). Treatment with BMD reduced both pro- and active MMP-9 (Figure 6l). A marked but not statistically significant reduction in MMP-9 was observed histologically in the *in vivo* A*β*-induced apoptosis model, although in the A*β*-treated eyes, the MMP-9 staining was significantly lower than in the no-OHT controls (*P*<0.001, Figure 6m). These results suggest that *α*2ARA modulate laminin and MMP-9 expression in RGCs, which may be linked to changes in A*β* and APP processing.

## Discussion

**Figure 7:**
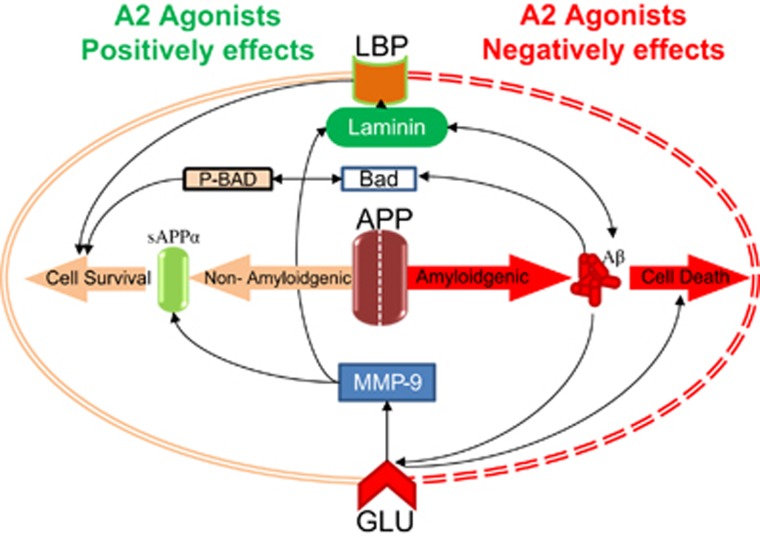
Diagram representing *α*2A agonists' neuroprotective effect against A*β* toxicity. APP can be processed into A*β* or sAPP*α* through the amyloidogenic and non-amyloidogenic pathways, respectively. *α*2A receptor agonists can negatively affect the amyloidogenic pathway, preventing cell death, partly by modulating excitotoxicity caused by glutamate (GLU). *α*2A receptor agonists can also affect APP processing via the extracellular matrix, by modulating MMP-9 and laminin through LBP, preventing further toxic interactions with A*β* and by increasing processing of APP into sAPP*α*, promoting the non-amyloidogenic pathway. *α*2A receptor agonists can also increase levels of P-Bad, and therefore overall act to promote cell survival and neuroprotection

The present study confirms the neuroprotective actions of *α*2ARAs using *in vivo* and *in vitro* models of retinal neurodegeneration with a novel IOP-independent mechanism of action. This mechanism proposes that a reduction in RGC apoptosis is achieved through reduced A*β* production, and its precursor APP, via stimulation of the non-amyloidogenic pathway as evidenced by a significant increase in sAPP*α,* which leads to modification of ECM proteins laminin and MMP-9 (Figure 7).

Compared with Clo, systemic BMD administration does not affect IOP,^31, 34^ suggesting that BMD's neuroprotective effect is IOP independent. This observation is supported by the LoGTs study,^13^ where BMD was effective in preserving visual field independent of IOP reduction in adults with low-pressure glaucoma. Further investigations of the clinical use of BMD as a neuroprotectant are currently underway (clinicaltrials.gov NCT00658619, NCT00693485, NCT01229410). Furthermore, the ability of BMD to improve visual function is documented in rodent models of retinal disease.^35, 36, 37^

This study suggests that different activity levels between *α*2ARAs could be attributed to differences in their selectivity for the *α*2A and the *α*2A_A_ subtype. BMD and Dex are direct agonists of the *α*2A_A_ receptor subtype, and are inhibited by the *α*2A_A_ antagonist yohimbine *in vivo*^27^ and *in vitro*.^38^ In comparison, Clo has reduced selectivity for *α*2 receptors.^8^^,17, 39^ Neuroprotective effects *α*2ARAs are understood to be primarily mediated though *α*2A receptors,^11^ supporting BMD's effects in this study. *α*2A receptors are G-protein-coupled-receptors whose activation leads to an inhibition of adenylate cyclase and a decrease in cAMP.^40^ cAMP is implicated in A*β* modulation by stimulation of APP synthesis and processing, affecting the amyloidogenic and non-amyloidogenic pathways,^41, 42^ potentially through the cAMP/PKA/APP/A*β* pathway.^43^

Several mechanisms have been proposed for *α*2A-mediated neuroprotection, including increased expression of neurotrophic factors.^17, 22, 27, 44, 45, 46^ Other survival pathways include P-Akt, bcl-2 and extracellular signal-regulated kinase (ERK),^27, 47^ which support this study's observed increase in P-Bad(Ser136). *α*2A agonists have also been reported to effect the glutamate pathway,^48^ with reductions in intracellular cAMP production and cytosolic calcium shown in RGCs.^21^ Recently, BMD was reported to reduce expression of NMDA subunits NR1 and NR2A in a model of ischaemia.^19^ Similar activity in excitotoxicity models is reported for Clo^17, 49^ and Dex^50^ in the CNS.

Elevated APP and A*β* have been reported in glaucoma models,^4, 51^ potentially linking elevated IOP promotion of APP processing, and inhibition of APP anterograde transport from the RGCL to the optic nerve.^51, 52^ BMD is reported to preserve optic nerve axons and active transport throughout the visual pathway in a rodent OHT model,^18^ which may explain the present study's finding where *α*2A agonists reduced levels of APP and A*β* in the RCGL of the OHT model. This hypothesis is supported by our *in vitro* data, where CoCl_2_ and UV-induced elevation of APP and A*β* was inhibited by *α*2ARAs. A possible pathway by which this may occur is through *α*2ARAs' ability to inhibit cAMP production.^21^

This study suggests for the first time that the neuroprotective effects of *α*2ARA BMD are directly associated with increased sAPP*α*, as the observed BMD-mediated neuroprotective effect was reversed using an sAPP*α* antibody *in vitro*. Furthermore, the increase in sAPP*α* appears to be specific to *α*2A_A_ activity, as Clo did not produce a comparable effect.^11^ Previously, sAPP*α* was reported to be neuroprotective *in vitro*.^53,29^ More recently, Obregon *et al.* reported that sAPP*α* decreases A*β* generation directly by associating with the *β*-site APP-converting enzyme (BACE1) both *in vivo* and *in vitro*. Importantly, the authors proposed that the levels of sAPP*α* were so crucial that an imbalance could stimulate amyloidogenic APP processing,^54^ concluding that sAPP*α* mimetics are a potential therapeutic target for the treatment of AD. This was further highlighted in a recent paper by Willem where unwanted side-effects on neuronal activity were implicated by treatment with BACE1.^55^ The results of the present study suggest that *α*2A_A_ agonists are sAPP*α* modulators, promoting RGC survival in a non-IOP-dependent manner.

A mechanism for the stimulation of sAPP*α* by *α*2A agonists may be through the activation of *α*-secretases, which can occur via protein kinase C (PKC), phosphatidylinositol 3-kinase (PI3K) and mitogen-activated protein kinase–ERK.^56^ Evidence suggests that APP cleavage by the *α*-secretase ADAM10 (a disintegrin and metalloproteinase and physiological *α*-secretase in neurons) constitutively produces sAPP*α* through 5-HT4 receptors in a cAMP-independent pathway. When stimulated by 5-HT4 receptors agonists, however, sAPP*α* secretion is mediated through cAMP/Epac (exchange protein activated by cAMP) signalling.^57^ A possible explanation for this study's findings is that *α*2ARAs could directly activate *α*-secretase ADAM10 and sAPP*α* production through cAMP/Epac modulation.

ADAM10 and MMP-9 have been reported to stimulate sAPP*α* production and decrease A*β*, through *α*-secretase-like activity.^58, 59^ We present *in vitro* data suggesting that *α*2ARAs increase levels of pro-MMP-9 and sAPP*α*, whereas decreasing A*β* levels. This concurs with *in vivo* studies where elevated MMP-9 levels in a A*β*PP transgenic mouse model led to a reduced plaque burden and increased sAPP*α* levels.^60^ As both ADAM10 and MMP-9 are matrix metalloproteases, their activity levels may be similar.^61, 62^ In the retina, NMDA activation is reported to increase MMP-9 activity;^63^ *α*2A agonists could therefore reduce MMP-9 activity by inhibiting retinal excitotoxicity.^21^ Furthermore, MMP-9 is reported to be influenced by A*β*, as intracerebroventricularly injected A*β* is reported to increase MMP-9 levels *in vivo*.^63^ The reduction in A*β* levels by *α*2ARAs observed may be responsible for the reported reduction in active MMP-9 levels. The effects of *α*2ARAs on MMP-9 appear complex and closely related: *α*2A agonists may reduce active MMP-9 levels through modulation of NMDA and A*β* activity, but this reduction in MMP-9 may in-turn decrease its *α*-secretase-like activity and the subsequent promotion of the non-amyloidogenic pathway.

MMP-9 activity in retinal neurodegeneration is linked with laminin, it was reported that RGC degeneration is associated with reduced laminin and stimulation of MMP-9.^33^ We report similar changes in laminin in the RGCL of the OHT model, but at earlier time points than previously described. Laminin is suggested to be neuroprotective through a laminin-integrin signalling pathway, with evidence that laminin promotes RGC survival *in vitro* through *β*1 integrin-focal adhesion kinase signalling.^64^ The reduction in laminin reported in this study was reversed by *α*2A agonist BMD treatment. BMD has been reported to induce laminin binding protein expression, laminin-induced neuronal and axonal changes, essential for promoting axonal preservation and growth.^65^

Furthermore, laminin is linked to modification of APP and A*β* processing and found in senile plaques in Alzheimer's Disease,^66^ where it prevents A*β* fibril formation and reduces neurotoxicity.^32^ This effect was confirmed *in vivo*, with laminin depletion accelerating A*β*-induced neurotoxicity, altering the distribution of A*β* aggregates in *Caenorhabditis* *elegans.*^67^ Intravitreal injection of A*β*_25–35_ was found to induce increased laminin in the RGCL, which was reversed by BMD treatment, contradictory to the effect of BMD treatment observed in the OHT model. The increase in laminin observed in the A*β*_25–35_ model may be a response to direct A*β*-induced neurotoxicity.^32, 67^ The reduction of A*β*-induced neurotoxicity with BMD treatment may result in an attenuation of the increased laminin levels in response to the acute presence of A*β*. This hypothesis is supported by the observation that BMD reduces levels of A*β* deposition in both the OHT and A*β* models *in vivo*, inhibiting A*β* neurotoxicity and RGC apoptosis.

In addition to modulating A*β* levels through sAPP*α*, this study confirms that *α*2A agonist BMD can elicit neuroprotection through P-Bad (Ser136) (Supplementary Figure1a–d). A*β* is reported to increase Bad de-phosphorylation and cell death by stimulating calmodulin–calcineurin activity.^68^ BMD-mediated reduction of A*β* may increase P-Bad, promoting cell survival pathways involving PI3K, known to be upregulated by BMD upstream of P-Bad.^27^ PI3K activity can itself decrease A*β* levels,^69^ providing further corroboration that BMD reduces A*β*. In addition, upstream of Bad, PKC activation simultaneously increases sAPP*α* and reduces A*β* levels, providing further support for non-amyloidogenic promotion of cell survival.^70^

An alternative and complementary pathway by which *α*2A agonists reduce A*β* expression in OHT, may be via oxidative stress modulation.^26^ Oxidative stress reduces expression of sAPP*α* and *α*-secretase activity^71^ and is implicated in the pathogenesis of glaucoma, where an ischaemic element is well-recognised.^72^ The observation that BMD increases sAPP*α* and reduces A*β* levels in RGCs when exposed to a hypoxic insult (CoCl_2_) supports evidence for *α*2A agonist's neuroprotective activity under oxidative stress. Although A*β*_1-42_ monomers may possess neuroprotective function,^6^ A*β* oligomers and A*β*_25–35_ used in this study are widely reported to induce oxidative stress.^4^ The effects of *α*2A agonists against oxidative stress are supported by our observation that BMD and Clo are protective against A*β*_25–35_-induced RGC apoptosis *in vivo*.

A*β* neurotoxicity is associated with glutamate NMDA receptor activation and is the basis of the FDA-approved NMDA receptor antagonist Memantine for the treatment of Alzheimer's Disease.^73^ This pathway has also been implicated in the development of A*β* induced-dendritic spine loss and tau-associated neurodegeneration.^74^ Short-term NMDA receptor stimulation is reported to increase non-amyloidogenic *α*-secretase-mediated APP processing and sAPP*α* production,^75^ whereas chronic stimulation is reported to have the opposite effect.^76^ *α*2ARAs can also modulate NMDA receptors; Dong *et al.* report that BMD acts post-synaptically on NMDA receptors by reducing levels of intracellular cAMP; this indirect effect could also be responsible for BMD suppressing A*β*-induced excitotoxicity.^21^ Clo has been shown to regulate GABAergic synaptic inputs in the CNS, which could explain its neuroprotective effect reported in this study in the OHT model.^49^

In conclusion, we confirm that *α*2ARAs are neuroprotective of RGC death *in vivo* and *in vitro*, substantiating previous reports that this effect is non-IOP dependent. We suggest a new mechanism by which this occurs; we show through multiple modalities that *α*2ARAs modulate A*β* toxicity, decreasing levels of A*β* and APP *in vivo* and *in vitro*, and increasing sAPP*α* formation through the non-amyloidogenic pathway (Figure 7). We specifically demonstrate that neuroprotective effects of *α*2ARA BMD are mediated through sAPP*α*. Furthermore, BMD affects laminin, in association with A*β*, which influences RGC survival. Recent studies have identified the need for *α*-secretase activators and sAPP*α*-mimetics in neurodegeneration. We propose that *α*2ARAs may be the most commonly available clinical sAPP*α* modulators, and being 'tried and tested' may offer an economical advantage as a pre-existing therapeutic for neuroprotection. The applications of *α*2ARAs may therefore not be limited to reducing RGC death in glaucoma but also to any neurodegenerative process where A*β* neurotoxicity is involved, such as AD. This work strongly advocates investigation of the therapeutic potential of *α*2ARAs in these disorders.

## Materials and Methods

### Ethics statement

All procedures were approved by the UK Home Office and the University College London Ethics Committee and were conducted in accordance with the Association for Research in Vision and Ophthalmology statement and ARRIVE guidelines.

### Animals

Adult male Dark Agouti rats (200–250 g) (*n*=50) were maintained in a 12-hour light/12-hour dark cycle, and provided standard food and water *ad libitum*. Animals were anaesthetised by intraperitoneal administration of ketamine (37.5%) (Ketaset; Fort Dodge Animal Health) and medetomidine (25%) (Dormitor; Pfizer, Exton, PA, USA) at 2 ml/kg, except for IOP measurements, for which animals were anaesthetised by 0.4% isoflurane in oxygen.

### Chemicals

BMD was purchased from Sigma Aldrich (Dorset, UK), or obtained from Allergan (Oregon, USA); Clo and Dex were purchased from Santa Cruz Biotechnology Inc (Santa Cruz, CA, USA).

### Rat model of OHT

The OHT model was surgically induced in 30 rats using a well-established method.^30^ IOP was elevated in the left eye of each animal by injection of 50 *μ*l hypertonic saline (1.80 M) into the episcleral veins using a syringe pump (UMP2, World Precision Instruments, Sarasota, FL, USA). A propylene ring, with a 1 mm gap cut out of its circumference, was placed around the equator to prevent saline outflow from other aqueous veins, as described previously.^4, 33^ Contralateral, un-operated eyes served as controls. The IOP of both eyes was measured in mmHg at regular intervals using a Tonolab Tonometer (Tiolat Oy, Heisinki, Finland). Treatments of BMD (*n*=10), Clo (*n*=10) and PBS control (*n*=10) were administered intraperitoneally at the time of surgery. Animals were imaged for RGC apoptosis by administrating Alexa-488-labelled annexin A5 (1.25 *μ*g in 5 *μ*l) intravitreally at 3 (*n*=15) and 8 (*n*=15) weeks, before being killed for histological assessment.^1, 33^ The ratio of IOP was calculated by dividing the IOP from the operated, OHT left eye (OS) by that of the un-operated, no-OHT right eye (OD). Analysts were masked to treatment group identity. Statistical analysis on IOP data was carried out using the non-parametric two-tailed Mann–Whitney test comparing treated OHT to untreated no-OHT control (*n*=5).

### Rat model of A*β*

The A*β* model was prepared in 15 rats to induce RGC apoptosis as described previously.^4^ In brief, 25nmol A*β*_25–35_ (Sigma Aldrich) was dissolved in sterile water and intravitreally administered unilaterally. BMD (*n*=5) Clo (*n*=5) or vehicle (PBS) (*n*=5) was administered intraperitoneally at the time of A*β* administration. Rats were imaged for RGC apoptosis (as described above) at baseline and 3 days following treatments before sacrifice.

### Histology and immunohistochemistry

RGC apoptosis counts were performed *in vivo* and histologically *ex vivo*, using whole-retina mounts.^4^ For whole-retina mounts, eyes were enucleated and immediately fixed in 4% paraformaldehyde following termination. Eyes were dissected at the equator, and the cornea, lens and vitreous were removed. RGC apoptosis was identified by fluorescent annexin A5 staining. RGC apoptosis density counts were performed in a masked fashion by three observers, and were calculated as previously described.^4, 77^

Immunohistochemistry was performed using antibodies listed in Table 1. Dissected eyes were fixed in 10% formalin before processing in methanol solutions with increasing concentrations, and embedding in paraffin.^33^ In total, 3 *μ*m thick sections were then cut and incubated with antibodies diluted in TBTA (Table 1). Stained sections were analysed using fluorescent microscopy (Zeiss Axiovert S100) at x20 magnification, by a masked observer. The RGCL was segmented using a box of fixed proportions and image background noise was subtracted using a 25 pixel rolling ball radius, before recording the mean fluorescence intensity through the pixels' grey values using ImageJ software (NIH) as previously described.^78^ Colocalisation analysis was carried out using the Pearson's Coefficient test with the ImageJ JaCoP plugin^79^ on segmented double-labelled RGCL images.

### Cell culture

Both primary murine retinal mixed neuronal cultures and an immortalised retinal neuronal (RN) cell line (a gift from Dr. Neeraj Agarwal, Department of Cell Biology and Genetics, UNT Health Science Centre, Fort Worth, TX, USA) were used. The immortalised line expresses retinal neuronal proteins Thy-1, Brn-3a and *β*3 tubulin^80^ and strong similarity to the 661w photoreceptor cell line.^81^ Immortalised cells were cultured in Dulbecco's modified Eagle's medium (DMEM; Sigma, Gillingham, UK), supplemented with 10% heat-inactivated fetal bovine serum (Life Technologies, Paisley, UK), 100 *μ*g/ml penicillin and 100 mg/ml streptomycin (Life Technologies). Primary murine (C57BL/6) mixed retinal cultures were isolated from P0 pups and neuronal cells isolated by incubation in a solution containing 10 units of papain/ml, and cultured in DMEM with 25 mM HEPES supplemented with 5% FCS, 15 mM KCl, x0.75 Penicillin/Streptovidin/glutamine (Gibco, Paisley, UK) and serum extender (BD Biosciences, Oxford, UK).

### Cell viability studies

The neuroprotective effects of *α*2 agonists were assessed *in vitro* using either the MTT or the alamarblue cell viability assays. In brief, RN were seeded at a density of 3 × 10^3^ cells/well in a 96-well plate and cultured for 24 h (37 °C, 5% CO_2_) before incubation with specified concentrations of BMD prepared from a 24 mM stock solution in DMSO, Clo and Dex for 24 h. Cells were insulted with predetermined (IC_50_) concentrations of CoCl_2_ or UV-B light for 24 h. Cell viability was assessed using MTT assay, where cells were treated with 0.5 mg/ml MTT in culture media for 2 h before dissolving the resulting formazan crystals using DMSO (0.1 ml/well) and measuring absorbance (Safire microplate reader) at 570 nm. For the alamarblue viability assay, 10 *μ*l of alamarblue solution was added per 100 *μ*l DMEM and incubated for 1.5 h measuring absorbance (Safire microplate reader) at 570nm. All experiments were carried out in triplicate.

### Assessment of secreted MMP-9 activity by zymography

RNs were cultured to 70–80% confluence before pretreatment with BMD and insulting with 250* μ*M CoCl_2_ for 24 h. Conditioned media was collected in the presence of protease cocktail inhibitors (Merck, Millipore, Nottingham, UK), and concentrated using centrifugal filters with a MWCO of 10kDa. Total protein concentration was determined using a BCA assay kit (Thermo Scientific, Rockford, IL, USA) according to manufacturer's instructions. Conditioned media containing 20 *μ*g of protein per sample was run on a 10% SDS gel containing 0.1% gelatin (Sigma) at 180 V for 1 h rtp. SDS was removed by two 45 min washes in 2.5% PBS-Triton, followed by incubation in developing buffer (Invitrogen, Paisley, UK) overnight at 37 °C. Gels were stained with Coomassie blue to reveal clear bands indicating the presence of secreted pro- and active MMP-9.^82^

### Immunocytochemistry

Immunocytochemistry was performed to elucidate mechanisms of *α*2 agonist-associated neuroprotection. RNs were seeded on glass coverslips before pre-treating with BMD, Clo or Dex for 24 h and insulted with 250 *μ*M CoCl_2_ or UV-B light (80 j/cm^2^) for a further 24 h. Cells were fixed in 4% paraformaldehyde before permeabilizing with 0.1% PBS-Triton and blocking with 3% BSA. Cells were incubated overnight in either anti-P-Bad (Ser136), anti-sAPP*α*, anti-APP or anti-A*β* with anti-*α* Tubulin (Cell Signalling Technology, Beverly, MA, USA) to visualise cell structure. Antibodies were diluted 1:100 in 3% BSA, except anti-*α* Tubulin (1:50) (Table 1). Fluorescence microscopy and analysis was performed as described previously. All experiments were carried out in triplicate.

### Experimental design

Sample size estimation for the OHT study was based on the size of 'neuroprotective' effects of BMD in glaucoma patients in preserving visual field in the LoGTs study.^13^ This effect was 90.9%, and based on a power of 0.85 and alpha of 5%, this provided a minimum number of four animals per group per time point. OHT rats were randomly assigned in blocks of three to treatment with either BMD or Clo (*n*=5 at 3 weeks and *n*=5 at 8 weeks), or vehicle (PBS, *n*=10). For the A*β* model, samples size was based on our previous published results.^4^ *Ex vivo* analyses were planned with five rats per group, but owing to one death and one atrophic retina, a final number of three to five rats per group were used, as sample material was limited. Animals were randomly assigned in blocks of three to treatment, as above. All *in vitro* experiments were carried out in triplicate.

### Statistical analysis

Unless otherwise stated all statistical tests comprise a one-way ANOVA with either Dunnett's or Tukey's post-tests, with *P*<0.05 taken as statistical significance.

## Figures and Tables

**Table 1:** Details of antibodies used for immunochemistry

**Antibody**	**Host**	**Dilution (for IHC)**	**Source**	**Secondary antibody and conjugated fluorophore**
A*β*	Rabbit	1:750	Abcam (ab68896)	Anti-rabbit Cy3
APP	Goat	1:1000	Abcam (ab2084)	Anti-goat FITC
MMP-9	Goat	1:400	Santa Cruz (sc-6840)	Anti-goat FITC
Laminin	Rabbit	1:50	Sigma (L9393)	Anti-rabbit Cy3
P-Bad (Ser136)	Rabbit	1:500	Signalway Antibody (11068)	Anti-rabbit Cy3
sAPP*α*	Rabbit	1:100	Covance (SIG-39139)	Anti-rabbit Cy3

## Notes

Professor MF Cordeiro holds a patent regarding DARC technology. The remaining authors declare no conflict of interest.

## References

[bib1] Cordeiro MF, Guo L, Luong V, Harding G, Wang W, Jones HE et al. Real-time imaging of single nerve cell apoptosis in retinal neurodegeneration. Proc Natl Acad Sci USA 2004; 101: 13352–13356. 15340151 10.1073/pnas.0405479101 PMC516570

[bib2] Quigley HA, Broman AT. The number of people with glaucoma worldwide in 2010 and 2020. Br J Ophthalmol 2006; 90: 262–267. 16488940 10.1136/bjo.2005.081224 PMC1856963

[bib3] Collaborative Normal-Tension Glaucoma Study. Comparison of glaucomatous progression between untreated patients with normal-tension glaucoma and patients with therapeutically reduced intraocular pressures. Collaborative Normal-Tension Glaucoma Study Group. Am J Ophthalmol 1998; 126: 487–497. 9780093 10.1016/s0002-9394(98)00223-2

[bib4] Guo L, Salt TE, Luong V, Wood N, Cheung W, Maass A et al. Targeting amyloid-beta in glaucoma treatment. Proc Natl Acad Sci USA 2007; 104: 13444–13449. 17684098 10.1073/pnas.0703707104 PMC1940230

[bib5] Goldblum D, Kipfer-Kauer A, Sarra G-M, Wolf S, Frueh BE. Distribution of amyloid precursor protein and amyloid-beta immunoreactivity in DBA/2J glaucomatous mouse retinas. Invest Ophthalmol Vis Sci 2007; 48: 5085–5090. 17962460 10.1167/iovs.06-1249

[bib6] Chasseigneaux S, Allinquant B. Functions of Aβ, sAPPα and sAPPβ: similarities and differences. J Neurochem 2012; 120: 99–108. 22150401 10.1111/j.1471-4159.2011.07584.x

[bib7] Yoneda S, Hara H, Hirata A, Fukushima M, Inomata Y, Tanihara H. Vitreous fluid levels of beta-amyloid(1-42) and tau in patients with retinal diseases. Jpn J Ophthalmol 2005; 49: 106–108. 15838725 10.1007/s10384-004-0156-x

[bib8] Burke J, Schwartz M. Preclinical evaluation of brimonidine. Surv Ophthalmol 1996; 41: S9–18. 8970245 10.1016/s0039-6257(96)82027-3

[bib9] Chen TC. Brimonidine 0.15% versus apraclonidine 0.5% for prevention of intraocular pressure elevation after anterior segment laser surgery. J Cataract Refract Surg 2005; 31: 1707–1712. 16246772 10.1016/j.jcrs.2005.02.035

[bib10] Chung HS, Shin DH, Birt CM, Kim C, Lee D, Levin DS et al. Chronic use of apraclonidine decreases its moderation of post-laser intraocular pressure spikes. Ophthalmology 1997; 104: 1921–1925. 9373127 10.1016/s0161-6420(97)30006-2

[bib11] Arthur S, Cantor LB. Update on the role of alpha-agonists in glaucoma management. Exp Eye Res 2011; 93: 271–283. 21524649 10.1016/j.exer.2011.04.002

[bib12] Cantor LB. The evolving pharmacotherapeutic profile of brimonidine, an alpha 2-adrenergic agonist, after four years of continuous use. Expert Opin Pharmacother 2000; 1: 815–834. 11249518 10.1517/14656566.1.4.815

[bib13] Krupin T, Liebmann JM, Greenfield DS, Ritch R, Gardiner S. A randomized trial of brimonidine versus timolol in preserving visual function: results from the Low-Pressure Glaucoma Treatment Study. Am J Ophthalmol 2011; 151: 671–681. 21257146 10.1016/j.ajo.2010.09.026

[bib14] Mantz J, Josserand J, Hamada S. Dexmedetomidine: new insights. Eur J Anaesthesiol 2011; 28: 3–6. 20881501 10.1097/EJA.0b013e32833e266d

[bib15] JOINT FORMULARY COMMITTEE. Joint Formulary Committee. British National Formulary (online) London: BMJ Group and Pharmaceutical Press http://www.medicinescomplete.com (accessed on 10 September 2016). British National Formulary. 2016 (cited 2016 September 10). Available from http://www.evidence.nhs.uk/formulary/bnf/current/4-central-nervous-system/47-analgesics/474-antimigraine-drugs/4742-prophylaxis-of-migraine/clonidine-hydrochloride.

[bib16] JOINT FORMULARY COMMITTEE. Joint Formulary Committee. British National Formulary (online) London: BMJ Group and Pharmaceutical Press http://www.medicinescomplete.com (accessed on 10 September 2016). British National Formulary. 2016 (cited 2016 September 10). Available from http://www.evidence.nhs.uk/formulary/bnf/current/15-anaesthesia/151-general-anaesthesia/1514-sedative-and-analgesic-peri-operative-drugs/15144-other-drugs-for-sedation/dexmedetomidine.

[bib17] Chao HM, Chidlow G, Melena J, Wood JP, Osborne NN. An investigation into the potential mechanisms underlying the neuroprotective effect of clonidine in the retina. Brain Res 2000; 877: 47–57. 10980242 10.1016/s0006-8993(00)02592-0

[bib18] Lambert WS, Ruiz L, Crish SD, Wheeler LA, Calkins DJ. Brimonidine prevents axonal and somatic degeneration of retinal ganglion cell neurons. Mol Neurodegener 2011; 6: 4. 21232114 10.1186/1750-1326-6-4 PMC3035592

[bib19] Lee D, Kim K-Y, Noh YH, Chai S, Lindsey JD, Ellisman MH et al. Brimonidine blocks glutamate excitotoxicity-induced oxidative stress and preserves mitochondrial transcription factor a in ischemic retinal injury. PLoS One 2012; 7: e47098. 23056591 10.1371/journal.pone.0047098 PMC3467218

[bib20] Chao HM, Osborne NN. Topically applied clonidine protects the rat retina from ischaemia/reperfusion by stimulating alpha(2)-adrenoceptors and not by an action on imidazoline receptors. Brain Res 2001; 904: 126–136. 11516418 10.1016/s0006-8993(01)02499-4

[bib21] Dong C-J, Guo Y, Agey P, Wheeler L, Hare WA. Alpha2 adrenergic modulation of NMDA receptor function as a major mechanism of RGC protection in experimental glaucoma and retinal excitotoxicity. Invest Ophthalmol Vis Sci 2008; 49: 4515–4522. 18566471 10.1167/iovs.08-2078

[bib22] Degos V, Charpentier T, Le, Chhor V, Brissaud O, Lebon S, Schwendimann L et al. Neuroprotective effects of dexmedetomidine against glutamate agonist-induced neuronal cell death are related to increased astrocyte brain-derived neurotrophic factor expression. Anesthesiology 2013; 118: 1123–1132. 23353792 10.1097/ALN.0b013e318286cf36

[bib23] Maier C, Steinberg GK, Sun GH, Zhi GT, Maze M. Neuroprotection by the alpha 2-adrenoreceptor agonist dexmedetomidine in a focal model of cerebral ischemia. Anesthesiology 1993; 79: 306–312. 8102042 10.1097/00000542-199308000-00016

[bib24] Schoeler M, Loetscher PD, Rossaint R, Fahlenkamp A V, Eberhardt G, Rex S et al. Dexmedetomidine is neuroprotective in an *in vitro* model for traumatic brain injury. BMC Neurol BioMed Central Ltd 2012; 12: 20. 10.1186/1471-2377-12-20 PMC3350422 22494498

[bib25] Woldemussie E, Wijono M, Pow D. Localization of alpha 2 receptors in ocular tissues. Vis Neurosci 2007; 24: 745–756. 17986363 10.1017/S0952523807070605

[bib26] Lee KYC, Nakayama M, Aihara M, Chen Y-N, Araie M. Brimonidine is neuroprotective against glutamate-induced neurotoxicity, oxidative stress, and hypoxia in purified rat retinal ganglion cells. Mol Vis 2010; 16: 246–251. 20161817 PMC2822551

[bib27] Lai RK, Chun T, Hasson D, Lee S, Mehrbod F, Wheeler L. Alpha-2 adrenoceptor agonist protects retinal function after acute retinal ischemic injury in the rat. Vis Neurosci 2002; 19: 175–185. 12385629 10.1017/s0952523802191152

[bib28] Tulsawani R, Kelly LS, Fatma N, Chhunchha B, Kubo E, Kumar A et al. Neuroprotective effect of peroxiredoxin 6 against hypoxia-induced retinal ganglion cell damage. BMC Neurosci 2010; 11: 125. 20923568 10.1186/1471-2202-11-125 PMC2964733

[bib29] Copanaki E, Chang S, Vlachos A, Tschäpe J-A, Müller UC, Kögel D et al. sAPPalpha antagonizes dendritic degeneration and neuron death triggered by proteasomal stress. Mol Cell Neurosci 2010; 44: 386–393. 20472066 10.1016/j.mcn.2010.04.007

[bib30] Morrison JC, Moore CG, Deppmeier LM, Gold BG, Meshul CK, Johnson EC. A rat model of chronic pressure-induced optic nerve damage. Exp Eye Res 1997; 64: 85–96. 9093024 10.1006/exer.1996.0184

[bib31] Weigert G, Resch H, Luksch A, Reitsamer Ha, Fuchsjager-Mayrl G, Schmetterer L et al. Intravenous administration of clonidine reduces intraocular pressure and alters ocular blood flow. Br J Ophthalmol 2007; 91: 1354–1358. 17537785 10.1136/bjo.2007.116574 PMC2000989

[bib32] Morgan C, Bugueño MP, Garrido J, Inestrosa NC. Laminin affects polymerization, depolymerization and neurotoxicity of Abeta peptide. Peptides 2002; 23: 1229–1240. 12128080 10.1016/s0196-9781(02)00058-x

[bib33] Guo L, Moss SE, Alexander RA, Ali RR, Fitzke FW, Cordeiro MF. Retinal ganglion cell apoptosis in glaucoma is related to intraocular pressure and IOP-induced effects on extracellular matrix. Invest Ophthalmol Vis Sci 2005; 46: 175–182. 15623771 10.1167/iovs.04-0832 PMC2601028

[bib34] Hernández M, Urcola JH, Vecino E. Retinal ganglion cell neuroprotection in a rat model of glaucoma following brimonidine, latanoprost or combined treatments. Exp Eye Res 2008; 86: 798–806. 18394603 10.1016/j.exer.2008.02.008

[bib35] Ferencz JR, Gilady G, Harel O, Belkin M, Assia EI. Topical brimonidine reduces collateral damage caused by laser photocoagulation for choroidal neovascularization. Graefes Arch Clin Exp Ophthalmol 2005; 243: 877–880. 15785924 10.1007/s00417-005-1160-7

[bib36] Evans DW, Hosking SL, Gherghel D, Bartlett JD. Contrast sensitivity improves after brimonidine therapy in primary open angle glaucoma: a case for neuroprotection. Br J Ophthalmol 2003; 87: 1463–1465. 14660453 10.1136/bjo.87.12.1463 PMC1920576

[bib37] Gandolfi SA, Sangermani C, Cimino L, Ungaro N, Tardini M, Viswanathan A et al. Is there a non-IOP related effect of brimonidine on visual field progression in human glaucoma? Invest Ophthalmol Vis Sci 2004; 45 ARVO E-Abstract 2298.

[bib38] Ma D, Hossain M, Rajakumaraswamy N, Arshad M, Sanders RD, Franks NP et al. Dexmedetomidine produces its neuroprotective effect via the alpha 2A-adrenoceptor subtype. Eur J Pharmacol 2004; 502: 87–97. 15464093 10.1016/j.ejphar.2004.08.044

[bib39] Virtanen R, Savola JM, Saano V, Nyman L. Characterization of the selectivity, specificity and potency of medetomidine as an alpha 2-adrenoceptor agonist. Eur J Pharmacol 1988; 150: 9–14. 2900154 10.1016/0014-2999(88)90744-3

[bib40] Maze M, Tranquilli W. Alpha-2 adrenoceptor agonists: defining the role in clinical anesthesia. Anesthesiology 1991; 74: 581–605. 1672060

[bib41] Kumar a, La Rosa FG, Hovland a R, Cole WC, Edwards-Prasad J, Prasad KN. Adenosine 3',5'-cyclic monophosphate increases processing of amyloid precursor protein (APP) to beta-amyloid in neuroblastoma cells without changing APP levels or expression of APP mRNA. Neurochem Res 1999; 24: 1209–1215. 10492515 10.1023/a:1020912704404

[bib42] Canepa E, Domenicotti C, Marengo B, Passalacqua M, Marinari UM, Pronzato M a et al. Cyclic adenosine monophosphate as an endogenous modulator of the amyloid-β precursor protein metabolism. IUBMB Life 2013; 65: 127–133. 23297063 10.1002/iub.1109

[bib43] Ricciarelli R, Puzzo D, Bruno O, Canepa E, Gardella E, Rivera D et al. A novel mechanism for cyclic adenosine monophosphate-mediated memory formation: Role of amyloid beta. Ann Neurol 2014; 75: 602–607. 24591104 10.1002/ana.24130

[bib44] Patel NJ, Chen MJ, Russo-Neustadt A a. Norepinephrine and nitric oxide promote cell survival signaling in hippocampal neurons. Eur J Pharmacol 2010; 633: 1–9. 20149790 10.1016/j.ejphar.2010.01.012

[bib45] Lönngren U, Näpänkangas U, Lafuente M, Mayor S, Lindqvist N, Vidal-Sanz M et al. The growth factor response in ischemic rat retina and superior colliculus after brimonidine pre-treatment. Brain Res Bull 2006; 71: 208–218. 17113948 10.1016/j.brainresbull.2006.09.005

[bib46] Wen R, Cheng T, Li Y, Cao W, Steinberg RH. Alpha 2-adrenergic agonists induce basic fibroblast growth factor expression in photoreceptors *in vivo* and ameliorate light damage. J Neurosci 1996; 16: 5986–5992. 8815881 10.1523/JNEUROSCI.16-19-05986.1996 PMC6579178

[bib47] Kim HS, Chang YI, Kim JH, Park CK. Alteration of retinal intrinsic survival signal and effect of alpha2-adrenergic receptor agonist in the retina of the chronic ocular hypertension rat. Vis Neurosci 2007; 24: 127–139. 17640403 10.1017/S0952523807070150

[bib48] Donello JE, Padillo EU, Webster ML, Wheeler LA, Gil DW. alpha(2)-Adrenoceptor agonists inhibit vitreal glutamate and aspartate accumulation and preserve retinal function after transient ischemia. J Pharmacol Exp Ther 2001; 296: 216–223. 11123383

[bib49] Li D-P, Atnip LM, Chen S-R, Pan H-L. Regulation of synaptic inputs to paraventricular-spinal output neurons by alpha2 adrenergic receptors. J Neurophysiol 2005; 93: 393–402. 15356178 10.1152/jn.00564.2004

[bib50] Jolkkonen J, Puurunen K, Koistinaho J, Kauppinen R, Haapalinna A, Nieminen L et al. Neuroprotection by the alpha2-adrenoceptor agonist, dexmedetomidine, in rat focal cerebral ischemia. Eur J Pharmacol 1999; 372: 31–36. 10374712 10.1016/s0014-2999(99)00186-7

[bib51] McKinnon SJ, Lehman DM, Kerrigan-Baumrind LA, Merges CA, Pease ME, Kerrigan DF et al. Caspase activation and amyloid precursor protein cleavage in rat ocular hypertension. Invest Ophthalmol Vis Sci 2002; 43: 1077–1087. 11923249

[bib52] Morin PJ, Abraham CR, Amaratunga A, Johnson RJ, Huber G, Sandell JH et al. Amyloid precursor protein is synthesized by retinal ganglion cells, rapidly transported to the optic nerve plasma membrane and nerve terminals, and metabolized. J Neurochem 1993; 61: 464–473. 7687653 10.1111/j.1471-4159.1993.tb02147.x

[bib53] Mattson MP, Cheng B, Culwell AR, Esch FS, Lieberburg I, Rydel RE. Evidence for excitoprotective and intraneuronal calcium-regulating roles for secreted forms of the beta-amyloid precursor protein. Neuron 1993; 10: 243–254. 8094963 10.1016/0896-6273(93)90315-i

[bib54] Obregon D, Hou H, Deng J, Giunta B, Tian J, Darlington D et al. Soluble amyloid precursor protein-α modulates β-secretase activity and amyloid-β generation. Nat Commun 2012; 3: 777. 22491325 10.1038/ncomms1781 PMC3520614

[bib55] Willem M, Tahirovic S, Busche MA, Ovsepian S V, Chafai M, Kootar S et al. η-Secretase processing of APP inhibits neuronal activity in the hippocampus. Nature 2015; 526: 443–447. 26322584 10.1038/nature14864 PMC6570618

[bib56] Thathiah A, De Strooper B. The role of G protein-coupled receptors in the pathology of Alzheimer's disease. Nat Rev Neurosci 2011; 12: 73–87. 21248787 10.1038/nrn2977

[bib57] Cochet M, Donneger R, Cassier E, Gaven F, Lichtenthaler SF, Marin P et al. 5-HT4 receptors constitutively promote the non-amyloidogenic pathway of APP cleavage and interact with ADAM10. ACS Chem Neurosci 2013; 4: 130–140. 23336052 10.1021/cn300095t PMC3547471

[bib58] Postina R. Activation of α-secretase cleavage. J Neurochem 2012; 120: 46–54. 10.1111/j.1471-4159.2011.07459.x 21883223

[bib59] Talamagas AA, Efthimiopoulos S, Tsilibary EC, Figueiredo-Pereira ME, Tzinia AK. Abeta(1-40)-induced secretion of matrix metalloproteinase-9 results in sAPPalpha release by association with cell surface APP. Neurobiol Dis 2007; 28: 304–315. 17761425 10.1016/j.nbd.2007.07.016

[bib60] Hashimoto G, Sakurai M, Teich AF, Saeed F, Aziz F, Arancio O. 5-HT_4_ receptor stimulation leads to soluble AβPPα production through MMP-9 upregulation. J Alzheimers Dis 2012; 32: 437–445. 22810092 10.3233/JAD-2012-111235 PMC8962674

[bib61] Geng L, Wang W, Chen Y, Cao J, Lu L, Chen Q et al. Elevation of ADAM10, ADAM17, MMP-2 and MMP-9 expression with media degeneration features CaCl2-induced thoracic aortic aneurysm in a rat model. Exp Mol Pathol 2010; 89: 72–81. 20621845 10.1016/j.yexmp.2010.05.006

[bib62] Kim YH, Jung JC. Suppression of tunicamycin-induced CD44v6 ectodomain shedding and apoptosis is correlated with temporal expression patterns of active ADAM10, MMP-9 and MMP-13 proteins in Caki-2 renal carcinoma cells. Oncol Rep 2012; 28: 1869–1874. 22923171 10.3892/or.2012.1986

[bib63] Mizoguchi H, Takuma K, Fukuzaki E, Ibi D, Someya E, Akazawa K et al. Matrix metalloprotease-9 inhibition improves amyloid beta-mediated cognitive impairment and neurotoxicity in mice. J Pharmacol Exp Ther 2009; 331: 14–22. 19587312 10.1124/jpet.109.154724

[bib64] Santos ARC, Corredor RG, Obeso BA, Trakhtenberg EF, Wang Y, Ponmattam J et al. β1 integrin-focal adhesion kinase (FAK) signaling modulates retinal ganglion cell (RGC) survival. PLoS One 2012; 7: e48332. 23118988 10.1371/journal.pone.0048332 PMC3485184

[bib65] Prokosch V, Panagis L, Volk GF, Dermon C, Thanos S. Alpha2-adrenergic receptors and their core involvement in the process of axonal growth in retinal explants. Invest Ophthalmol Vis Sci 2010; 51: 6688–6699. 20592227 10.1167/iovs.09-4835

[bib66] Murtomäki S, Risteli J, Risteli L, Koivisto UM, Johansson S, Liesi P. Laminin and its neurite outgrowth-promoting domain in the brain in Alzheimer's disease and Down's syndrome patients. J Neurosci Res 1992; 32: 261–273. 1404496 10.1002/jnr.490320216

[bib67] Jensen LT, Møller TH, Larsen SA, Jakobsen H, Olsen A. A new role for laminins as modulators of protein toxicity in Caenorhabditis elegans. Aging Cell 2012; 11: 82–92. 22051349 10.1111/j.1474-9726.2011.00767.x PMC3257398

[bib68] Reese LC, Zhang W, Dineley KT, Kayed R, Taglialatela G. Selective induction of calcineurin activity and signaling by oligomeric amyloid beta. Aging Cell 2008; 7: 824–835. 18782350 10.1111/j.1474-9726.2008.00434.x PMC2954114

[bib69] Lee K-Y, Koh S-H, Noh MY, Kim SH, Lee YJ. Phosphatidylinositol-3-kinase activation blocks amyloid beta-induced neurotoxicity. Toxicology 2008; 243: 43–50. 17980476 10.1016/j.tox.2007.09.020

[bib70] Yang H-Q, Pan J, Ba M-W, Sun Z-K, Ma G-Z, Lu G-Q et al. New protein kinase C activator regulates amyloid precursor protein processing *in vitro* by increasing alpha-secretase activity. Eur J Neurosci 2007; 26: 381–391. 17650113 10.1111/j.1460-9568.2007.05648.x

[bib71] Quiroz-Baez R, Rojas E, Arias C. Oxidative stress promotes JNK-dependent amyloidogenic processing of normally expressed human APP by differential modification of alpha-, beta- and gamma-secretase expression. Neurochem Int 2009; 55: 662–670. 19560504 10.1016/j.neuint.2009.06.012

[bib72] Tezel G. The immune response in glaucoma: a perspective on the roles of oxidative stress. Exp Eye Res 2011; 93: 178–186. 20709058 10.1016/j.exer.2010.07.009 PMC2998544

[bib73] Danysz W, Parsons CG. Alzheimer's disease, β-amyloid, glutamate, NMDA receptors and memantine—searching for the connections. Br J Pharmacol 2012; 167: 324–352. 22646481 10.1111/j.1476-5381.2012.02057.x PMC3481041

[bib74] Tackenberg C, Grinschgl S, Trutzel A, Santuccione a C, Frey MC, Konietzko U et al. NMDA receptor subunit composition determines beta-amyloid-induced neurodegeneration and synaptic loss. Cell Death Dis 2013; 4: e608. 23618906 10.1038/cddis.2013.129 PMC3641351

[bib75] Hoey SE, Williams RJ, Perkinton MS. Synaptic NMDA receptor activation stimulates alpha-secretase amyloid precursor protein processing and inhibits amyloid-beta production. J Neurosci 2009; 29: 4442–4460. 19357271 10.1523/JNEUROSCI.6017-08.2009 PMC6665739

[bib76] Lesné S, Ali C, Gabriel C, Croci N, MacKenzie ET, Glabe CG et al. NMDA receptor activation inhibits alpha-secretase and promotes neuronal amyloid-beta production. J Neurosci 2005; 25: 9367–9377. 16221845 10.1523/JNEUROSCI.0849-05.2005 PMC6725703

[bib77] Salt TE, Nizari S, Cordeiro MF, Russ H, Danysz W. Effect of the Aβ aggregation modulator MRZ-99030 on retinal damage in an animal model of glaucoma. Neurotox Res 2014; 26: 440–446. 25106883 10.1007/s12640-014-9488-6

[bib78] Hoh Kam J, Lenassi E, Jeffery G. Viewing ageing eyes: diverse sites of amyloid Beta accumulation in the ageing mouse retina and the up-regulation of macrophages. PLoS One 2010; 5: e13127. 20957206 10.1371/journal.pone.0013127 PMC2948519

[bib79] Cordeiro M, Guo L, Coxon K, Duggan J. Realtime imaging of retinal ganglion cell apoptosis. Eur Ophthalmic Rev 2010; 4: 88–91.

[bib80] Galvao J, Davis B, Tilley M, Normando E, Duchen MR, Cordeiro MF. Unexpected low-dose toxicity of the universal solvent DMSO. FASEB J 2014; 28: 1317–1330. 24327606 10.1096/fj.13-235440

[bib81] Van Bergen NJ, Wood JPM, Chidlow G, Trounce I a, Casson RJ, Ju W-K et al. Recharacterization of the RGC-5 retinal ganglion cell line. Invest Ophthalmol Vis Sci 2009; 50: 4267–4272. 19443730 10.1167/iovs.09-3484

[bib82] Guo L, Hussain AA, Limb GA, Marshall J. Age-dependent variation in metalloproteinase activity of isolated human Bruch's membrane and choroid. Invest Ophthalmol Vis Sci 1999; 40: 2676–2682. 10509665

