# Supplementary figures and images for: Non-amyloidogenic effects of α2 adrenergic agonists: implications for brimonidine-mediated neuroprotection
Source: Cell Death Dis. 2016 Dec 8;7(12):e2514–. doi: 10.1038/cddis.2016.397 (PMC5260990; doi:10.1038/cddis.2016.397)

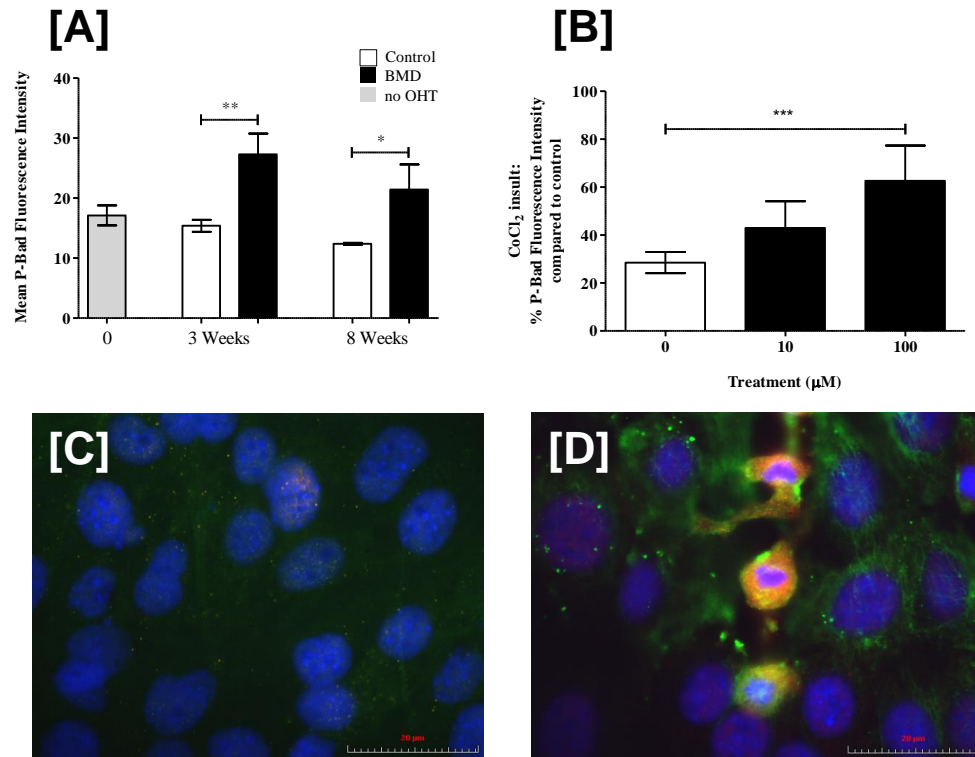

**Suppl Figure 1**

Supplement: Supplementary Figure 1 [file cddis2016397x1.pdf]
